# An Electrostatically Crosslinked Chitosan Hydrogel as a Drug Carrier

**DOI:** 10.3390/molecules171213704

**Published:** 2012-11-22

**Authors:** Ga On Kim, Nawoo Kim, Da Yeon Kim, Jin Seon Kwon, Byoung-Hyun Min

**Affiliations:** 1 Hankuk Academy of Foreign Studies, Yongin 449-854, Korea; E-Mails: gaon1995@gmail.com (G.O.K.); knw1225@gmail.com (N.K.); 2 Department of Molecular Science and Technology, Ajou University, Suwon 443-749, Korea; E-Mails: dayeon@ajou.ac.kr (D.Y.K.); jinseon@ajou.ac.kr (J.S.K.)

**Keywords:** chitosan, glycerol phosphate disodium salt, hydrogel, protein, depot

## Abstract

Considerable efforts have been devoted to control and maintain the sustained release of proteins. In this experiment, we used bovine serum albumin-fluorescein isothiocyanate (BSA-FITC) as a model protein to explore the potential utility of a chitosan and glycerol phosphate disodium salt (GP) hydrogel as a protein drug depot. The mixing of chitosan and GP solutions (0, 10, 20 and 30 wt%) formed a liquid at room temperature. At 37 °C, however, the chitosan/GP solutions formed hydrogels through an electrostatic crosslinking process. This electrostatic interaction between the chitosan, cationic amine group, and GP, anionic phosphate group, was confirmed by the changes of zeta potentials and particle sizes of this solution. The electrostatic interaction depended both on the GP ratios in chitosan and the incubation time of chitosan/GP solutions. Furthermore, BSA-FITC-loaded chitosan/GP hydrogels were examined for their ability as potential depots for the BSA drugs. Hence, when observed, the BSA-FITC-loaded chitosan/GP hydrogels showed an *in vitro* sustained release profile of BSA up to 14 days. Collectively, our results show that the chitosan/GP hydrogels described here, can serve as depots for BSA drugs.

## 1. Introduction

Most therapeutic protein drugs are currently administered in the form of oral pills or intravenous injections [1]. However, since protein drugs exhibit poor bioavailability, the administration of these drugs through these routes has a number of disadvantages: failure to achieve long-term *in vivo* release or unacceptably transient high or low plasma concentrations that may lead to toxicity [2]. Thus, this leads to the increased frequency of protein administration and affects patient compliance. Based on these reasons, an administration system to address these drawbacks is needed.

Recently considerable attention has been focused on drug delivery systems capable of local release of protein drugs for predefined periods [3]. Among the methods that have been developed, a hydrogel drug depot has been proven to both sustain protein release and improve protein stability [4,5]. Various hydrogel drug depots such as collagen, hyaluronate, and fibrin, as well as synthetic materials like Pluronic^®^ and various block copolymers, have been proposed for the same reason [6]. Likewise, chitosan, an amino-polysaccharide obtained by alkaline deacetylation of chitin, is an abundant natural polymer. Hence, chitosan-based hydrogels are considered as the appropriate candidate material for protein drugs [7,8,9,10,11,12].

**Figure 1:**
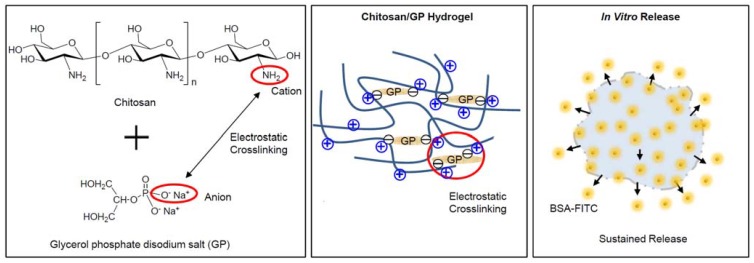
Schematic image of chitosan/GP hydrogel formation via electrostatic crosslinking between amine cationic groups of the chitosan chains and the phosphate anionic groups of the GP, and *in vitro* release of BSA-FITC from BSA-FITC-loaded chitosan/GP hydrogels.

Electrostatic crosslinking occurs due to molecular interactions between cationic and anionic polyelectrolytes. The cationic amine group of chitosan and anionic phosphate group of GP can induce electrostatic interactions and can create electrostatic crosslinking to make chitosan-based hydrogels (Figure 1). In addition, the formation of chitosan hydrogels can be affected by the increasing and decreasing interchain bonding of chitosan under variable temperatures. Recent studies have shown that a mixture of chitosan and glycerol phosphate disodium salt (GP) is capable of forming hydrogels through the process of electrostatic interaction [13,14].

The aim of this work was to examine the capability of chitosan/GP hydrogels to serve as depots for protein drugs. Thus, we first examined the sol-to-gel phase transition of chitosan/GP solutions, reflecting the electrostatic interaction between the cationic group of chitosan and anionic group of GP. Our second aim was to examine the *in vitro* release from a chitosan/GP hydrogel of bovine serum albumin-fluorescein isothiocyanate (BSA-FITC), as a model protein. The aims described here will contribute to the development of chitosan/GP hydrogels for drug delivery system.

## 2. Results and Discussion

### 2.1. Formation of Chitosan/GP Hydrogel

Each chitosan solution and GP solution exhibited a clear and homogeneous form. First, the zeta potential and particle size of GP only solution, chitosan only solution (CGP-0), chitosan/GP (10 wt% GP) (CGP-10), chitosan/GP (20 wt% GP) (CGP-20) and chitosan/GP (30 wt% GP) (CGP-30) at 37 °C were measured (Table 1) to confirm the electrostatic interaction between the cationic amine group of chitosan and anionic phosphate group of GP as shown in Figure 1.

The zeta potentials of GP solution and chitosan solution were −50 mV and +51 mV, respectively. The values were assigned to the anionic phosphate group of GP and cationic amine group of chitosan. The 51 mV zeta potential of the cationic group of chitosan decreased to 14 mV (CGP-10), 5 mV (CGP-20) and to 4 mV (CGP-30) as the anionic group of the GP was added to each of the chitosan solutions. This indicated that the chitosan interacted electrostatically with GP which in turn decreased the positive charges of chitosan to form chitosan/GP particles. Also, particle sizes increased from 400 nm in chitosan only solution, to 430 nm, 600 nm and 1,070 nm with the increase of GP concentration. These results indicated that the chitosan aggregated electrostatically with GP to form a more assembled chitosan solution.

These results proved the electrostatic interactions between the chitosan and the GP occur, and in addition implied the fact that the strength of the electrostatic interactions depends on the concentration of the anionic phosphate group to the cationic amine group.

**Figure 2:**
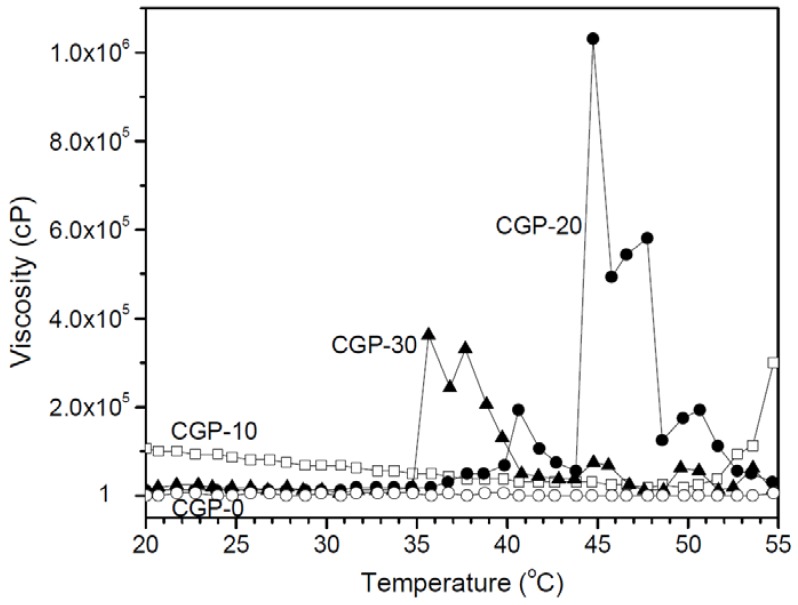
Viscosity *versus* temperature curves for CGP-0, CGP-10, CGP-20 and CGP-30.

Next, we examined the temperature-dependent viscosity changes of the CGP-0, CGP-10, CGP-20 and CGP-30 solutions. Viscosity was plotted at temperatures ranging from 20 to 55 °C as shown in Figure 2. Through the results it is shown that CGP-0 has no viscosity change during the increase in temperature. Proportional to the temperature, CGP-10 shows little viscosity change and its values indicate very low viscosity. The viscosity changed 1 × 10^5^–1.5 × 10^4^ cP below 45 °C to 1 cP above 45 °C, displaying a typical gel-to-sol phase transition. Moreover, CGP-10 shows a viscosity value of 3.8 × 10^4^ cP at 37 °C. We believed that the viscosity may depend on the balance between electrostatic attraction of chitosan and GP, and electrostatic repulsion in the interchain of chitosan as the temperature changed. In the present work, the viscosity behavior of CGP-10 is due to the increased electrostatic interchain repulsion of the chitosan.

On the other hand, CGP-20 and CGP-30 shows definite viscosity changes depending on temperature. The viscosities of CGP-20 and CGP-30 are close to those of water at temperatures below 37 °C and 34 °C respectively, likely reflecting little or no electrostatic interactions between the amine group of chitosan and the phosphate group of GP. When CGP-20 and CGP-30 reached 40 °C and 36 °C, it marks an increase in the viscosity. CGP-20 shows viscosity of 5.0 × 10^4^ cP at 37 °C and CGP-30 shows viscosity of 3.3 × 10^5^ cP at 37 °C. Meanwhile, CGP-20 showed maximum viscosity of 1.1 × 10^6^ cP at 45 °C. This displays the typical sol-to-gel phase transition of CGP-20 and CGP-30 due to the electrostatic interactions between the different ionic groups of GP and chitosan or the decreased electrostatic interchain repulsion of the chitosan.

**Figure 3:**
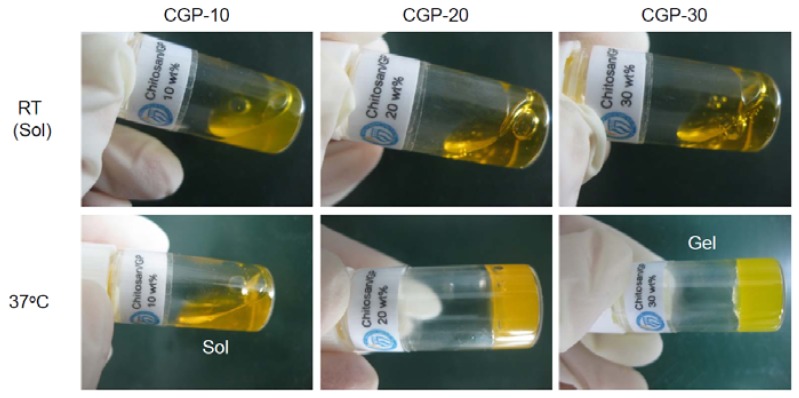
Images of BSA-FITC-loaded CGP-10, CGP-20 and CGP-30 at room temperature and 37 °C after 4 h.

To examine the potential of the CGP-10, CGP-20 and CGP-30 solutions as hydrogel depots, we prepared each of the different GP concentration chitosan solutions containing BSA-FITC. The BSA-FITC-loaded CGP-10, CGP-20 and CGP-30 remained in liquid state at room temperature (Figure 3). Of those three samples, the BSA-FITC-loaded CGP-10 exhibited a slightly more viscous liquid form compared to that of CGP-20 and CGP-30. When these solutions were immersed in a 37 °C water bath for 20 min, 4 h and 8 h and tilted to observe the solidification to hydrogels, the CGP-10 remained in liquid state at 37 °C after 20 min and its state did not change even after 8 h. On the other hand, CGP-20 did not form a hydrogel at 37 °C after 20 min, but formed one after 4 h at 37 °C. These results implied that the electrostatic interactions depended on the incubation time. Meanwhile, CGP-30 formed a hydrogel at 37 °C after 20 min. In addition, the formed CGP-30 hydrogel maintained its hydrogel form for the following four weeks *in vitro* (data not shown).

### 2.2. *In Vitro* BSA Release

**Figure 4:**
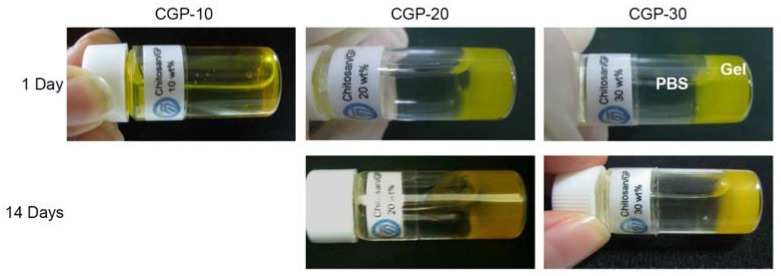
Images of BSA-FITC-loaded CGP-10, CGP-20 and CGP-30 at 37 °C after the addition of 37 °C PBS.

First, to test the persistence of the gel structure in an aqueous environment, we added a PBS solution (the temperature being 37 °C) to the BSA-FITC-loaded CGP-10, CGP-20 and CGP-30 after the incubation at 37 °C for 8 h (Figure 4). The BSA-FITC-loaded CGP-10 became a solution at 37 °C after the addition of PBS. The BSA-FITC-loaded CGP-10 hydrogel, on the other hand, dissolved due to the dissolution of the electrostatic interaction in chitosan. Meanwhile, the CGP-20 and CGP-30 hydrogels maintained its gel form for at least 14 days, even after the addition of PBS. 

**Figure 5:**
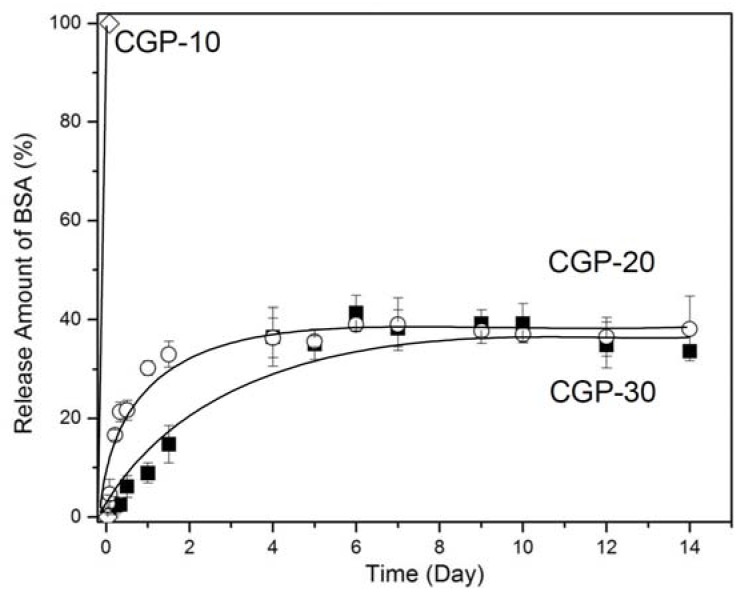
Cumulative amount of BSA-FITC released *in vitro* from BSA-FITC-loaded CGP-10, CGP-20 and CGP-30 over 14 days.

The cumulative amount of BSA-FITC released from CGP-10, CGP-20 and CGP-30 is shown in Figure 5. CGP-10 exhibited almost a complete release of BSA within 10 min, because the solution was completely dissolved after the addition of 37 °C PBS. Thus, the CGP-20 and CGP-30 solutions were examined for the *in vitro* BSA release experiment. The CGP-20 and CGP-30 exhibited the *in vitro* BSA-FITC release profile over 14 days. After 24 h, approximately 35% of the protein had been released from the CGP-20 hydrogel, indicating an initial burst of BSA-FITC from the CGP-20 hydrogel. CGP-30 hydrogel, on the other hand, showed the release of approximately 10% BSA at 24 h, indicating that the CGP-30 hydrogel suppressed any initial burst of BSA-FITC. Approximately 40% of the BSA-FITC had been released from the CGP-20 and CGP-30 hydrogels after 4 days and 6 days, respectively. The release amount was maintained for 14 days. This indicated that the CGP-20 and CGP-30 hydrogels sustained the BSA-FITC release for 14 days.

## 3. Experimental

### 3.1. Preparation of Chitosan Solution with and without GP

The chitosan solution was obtained by dissolving chitosan (400 mg, medium viscosity, 75–80% deacetylated; Aldrich, St Louis, MO, USA) in 0.1 M acetic acid solution (18 mL) prepared in phosphate buffered saline (PBS). Then, GP (1 g) was also dissolved in PBS (1 mL). To produce the desired mixtures, the appropriate GP solution, 0 mL, 0.4 mL, 1 mL and 1.7 mL, was added dropwise to chitosan (4 mL) to yield 0 wt% GP (CGP-0), 10 wt% GP (CGP-10), 20 wt% GP (CGP-20) and 30 wt% GP (CGP-30), respectively. Each final liquid solution was clear and homogeneous.

### 3.2. Characterization of the Interaction between Chitosan and GP Solution

The prepared CGP-0, CGP-10, CGP-20 and CGP-30 solutions as described in the previous sections were diluted thirty-fold using deionized water. The CGP-0, CGP-10, CGP-20 and CGP-30 solutions interacted for 30 min before the measurements. The zeta potentials and particle sizes of the chitosan/GP solutions were determined at 37 °C by dynamic light scattering (DLS, ELS Z, Otsuka Electronics, Osaka, Japan). 

### 3.3. Determination of the Sol-to-Gel Transition

The CGP-0, CGP-10, CGP-20 and CGP-30 solutions were prepared in 5-mL vials with deionized water and were stored at 4 °C. The phase transitions for the sol and gel of each chitosan/GP solution was determined by tilting the vial at room temperature and 37 °C, respectively. The vials at room temperature and 37 °C were kept in a water bath for 20 min, 4 h and 8 h each, before tilting. The formation of a chitosan/GP hydrogel was displayed when the chitosan/GP solution did not flow as the vial tilted.

### 3.4. Viscosity Measurements

Viscosity of the solutions was measured using a Brookfield Viscometer DV-III Ultra with a programmable rheometer, circulating bath, and a programmable controller (TC-502P). In the viscosity measurement a tight cap was used to prevent the evaporation of water from the CGP-0, CGP-10, CGP-20 and CGP-30 solutions. The viscosities of the chitosan/GP solutions were examined using a T-F spindle at 0.2 rpm from 10–60 °C with the 2 °C increments.

### 3.5. In Vitro BSA-FITC Release

Since CGP-0 maintained its sol state at 37 °C, the *in vitro* BSA-FITC was only examined for CGP-10, CGP-20 and CGP-30. One-milliliter of each of the CGP-10, CGP-20 and CGP-30 solutions, containing BSA-FITC (1 mg/mL), were prepared in separate 5-mL vials and then incubated at 37 °C for 8 h. Then, 37 °C PBS (4 mL) was added to the CGP-10, CGP-20 and CGP-30 hydrogel to start the *in vitro* release observation. The vial was shaken constantly at 37 °C 100 rpm to determine the release of the protein. At specified times, a 1-mL sample of the solution was collected and fresh PBS (1 mL) at 37 °C was added to the vial. The removed sample was analyzed by fluorescence spectroscopy (F-6500, Jasco, Tokyo, Japan). The amount of cumulatively released BSA was calculated by reference to a standard calibration curve prepared by measuring the fluorescence of solutions containing known concentrations of BSA–FITC in PBS. Three independent release experiments were performed for the hydrogel compositions.

## 4. Conclusions

Throughout experiments, we explored the potential utility of a chitosan/GP hydrogel as a BSA drug depot. We showed that BSA-FITC-loaded chitosan/GP solutions at room temperature solidified at 37 °C through electrostatic interactions between the anionic group of GP and the cationic group of chitosan. We also demonstrated the sustained release of BSA-FITC from the chitosan/GP gel *in vitro* over 14 days. Based on these results we conclude that chitosan/GP hydrogels may provide numerous benefits as therapeutic drug depots and act as useful experimental platforms for testing the sustained pharmacological performance of protein drugs. Further study for a quantitative determination of the crosslinking according to the mole ratios of chitosan amines to GP to impact the sol-to-gel process and the complete release of BSA from chitosan/GP hydrogels are needed as future studies.

## Figures and Tables

**Table 1:** Zeta potentials and particle sizes of GP only solution, chitosan only solution, CGP-10, CGP-20 and CGP-30 at 37 °C.

Solutions	Zeta Potential (mV)	Particle Size (nm)
GP only solution	−49.9 ± 17.1	ND
Chitosan only solution (CGP-0)	51.3 ± 36.7	400 ± 36
CGP-10	13.6 ± 2.0	427 ± 3
CGP-20	5.3 ± 2.0	597 ± 51
CGP-30	3.6 ± 0.3	1070 ± 22

ND: not determined.
